# A moderated mediation model to predict the adoption intention of renewable wind energy in developing countries

**DOI:** 10.1371/journal.pone.0281963

**Published:** 2023-03-02

**Authors:** Sohaib Mustafa, Wen Zhang, Muhammad Tayyab Sohail, Sehrish Rana, Ying Long

**Affiliations:** 1 College of Economics and Management, Beijing University of Technology, Beijing, PR. China; 2 School of Public Administration, Xiangtan University, Xiangtan, PR. China; 3 Government Islamia Graduate College for Women, Faisalabad, Pakistan; 4 Beijing University of Technology, Beijing, PR. China; East China Normal University, CHINA

## Abstract

Developing countries are deprived of resources, and their economies are dwindling. Energy shortfall is one of the leading issues in developing countries that not only shatter economies but is the leading cause of depletion of natural resources and environmental pollution. There is an urgent need to shift to renewable energy sources to save economies and natural resources and to save our ecological system. Aiming for this, we have collected a cross-sectional data set to study the household intentions of shifting to wind energy and analysed the moderated mediation interactions of the variable to better understand socio-economic and personal factors. 840 responses were analysed using smart-PLS 4.0; results revealed that cost value and social influence directly relate to renewable energy adoption. Environmental knowledge directly influences attitude towards the environment, and health consciousness influences perceived behavioural control. Results also revealed that social influence strengthens the indirect relationship between awareness of renewable energy and its adoption, while it weakens the indirect relationship between health consciousness and renewable energy adoption.

## Introduction

Energy is a fundamental factor in the expansion of any nation’s economy and the achievement of long-term sustainable development. Many developing nations, as well as rich countries, are shifting their focus toward alternate renewable energy sources such as wind energy, solar energy, and biomass in order to meet the rapidly growing demand for energy, the depletion of fossil fuels, and worries about the environment. Wind power as a kind of renewable energy is now receiving a great deal of focus and consideration. Wind energy is a sustainable way to create energy that has the potential advantages of being a clean source of energy, reducing emissions of hazardous gases, and operating in a manner that is favourable to the environment.

The majority of developing countries are struggling with their dwindling economies, and a major chunk of their budgets goes into the purchase of oil and other energy sources that are not green [1, 2]. On one side, it costs a lot of money; on the other, it pollutes the environment. Developing countries must shift to renewable green energy sources and reduce their spending on crude oil and related energy sources. Hence, by understanding the influence of environmental knowledge, environmental concern, health consciousness, and awareness about renewable energy sources on domestic users, It will be easier for the government and other supporting agencies to shift from traditional energy sources to renewable energy sources. Self-reliance and using renewable energy at the domestic level will help revive dwindling economies in developing countries and reduce greenhouse gas emissions.

Wind power has the ability to solve all of our energy problems since it is not dependent on oil in any way, making it immune to the ups and downs of the oil market. It is also clean and favourable to the environment, and it is an innovative technology that will assist us in averting future environmental catastrophes. It is predicted that Pakistan has an electricity shortfall of 38.36 terawatt-hours, and the country’s wind and solar energy potential may enable it to meet 86% of that gap [3]. The generation of electricity by wind does not result in the emission of harmful pollutants into the atmosphere, which helps improve air quality. It has stringent regulations for environmental responsibility. On the one hand, the wind is an almost limitless resource.

It is impossible to ignore wind turbines’ manufacturing, installation, and transportation aspects. However, once the technology is put into place, there will no longer be any emissions. As a direct consequence of this, it is very healthy for the environment. In contrast to thermal power production, the generation of renewable energy by wind power stations results in no direct emission of greenhouse gases while also contributing to the advancement of sustainable development.

Pakistan has a lot of untapped potential for renewable energy sources; nevertheless, the country’s current production is pathetic. While solar power accounts for just 1.16% of the country’s overall electrical output, fossil fuels make up 64% of the total. Hydroelectricity contributes 27% of the country’s total electricity output, whereas nuclear power contributes just 5%. There is just a 4% contribution from renewable energy sources to the entire production of power [3, 4].

Recent studies have investigated renewable energy adoption and focused on financial literacy [5], state ownership [6], social networking to adopt biogas in china [7], and The importance of prioritising measures to remove obstacles to the expansion and use of renewable energy sources [8], cognitive abilities [9], Modularity in policy priority setting and the rate of renewable energy adoption [10], adoption delay [11] Local leader’s influence [12], consumers’ protected values and perceived comfort [13], and government support [14]. Still, researchers have ignored the influence of environmental knowledge, environmental concern, health consciousness, and awareness about renewable energy sources and potential. Furthermore, researchers have ignored the moderation effect of social influence on the mediation between the above-mentioned factors and renewable energy adoption.

The majority of these studies are conducted in developed countries, and there is a rift in studying the developing countries’ adoption intention towards renewable energy. Despite the fact that big industrial countries are responsible for greenhouse gas emissions, the fact of low income to buy fossil fuels for energy production is high in developing countries, and ecological damage awareness is less in developing countries. We believe that if the adoption of renewable energy is encouraged in developing countries, it can somehow help to control the ecological damage caused by greenhouse gas emissions. Hence we have selected Pakistan as one of the developing countries and the fifth largest population to study the household intention to adopt renewable energy sources.

Based on this research gap, we have proposed the following research questions.

**RQ1:** What is the influence of perceived behavioural control and its antecedents on renewable energy adoption in developing countries?

**RQ2:** What is the influence of attitudes toward the environment and its antecedents in renewable energy adoption in developing countries?

**RQ3:** Does social influence moderates the mediation role of attitudes toward the environment and perceived behavioural control between their antecedents and renewable energy adoption in developing countries?

To answer these research questions, we have proposed a model based on the theory of planned behaviour. We have collected a cross-sectional dataset from Sukhhur, Pakistan, and used smart PLS 4.0 software to analyse the moderated mediation model. Study results revealed that Health consciousness (HC) influences perceived behavioural control (PBC), and Environmental knowledge (EK) influences an attitude toward the environment (ATE). We further explored that perceived behavioural control and attitude towards the environment mediate the relationship between health consciousness, environmental knowledge, and renewable energy adoption. Results also elaborated that social influence moderates the mediation relationship of perceived behaviour control for Health consciousness and awareness of renewable energy.

Developing nations may benefit economically from a greater reliance on renewable energy resources if they are aware of the attitudes and intentions of potential domestic consumers over their adoption of such technologies. Developing nations may reduce their reliance on foreign oil and carbon footprint by installing renewable energy facilities and using their indigenous renewable energy sources better. As a result, the faltering economies may be stabilised, and the international community can take action to counteract climate change.

## Theoretical framework

**Fig 1:**
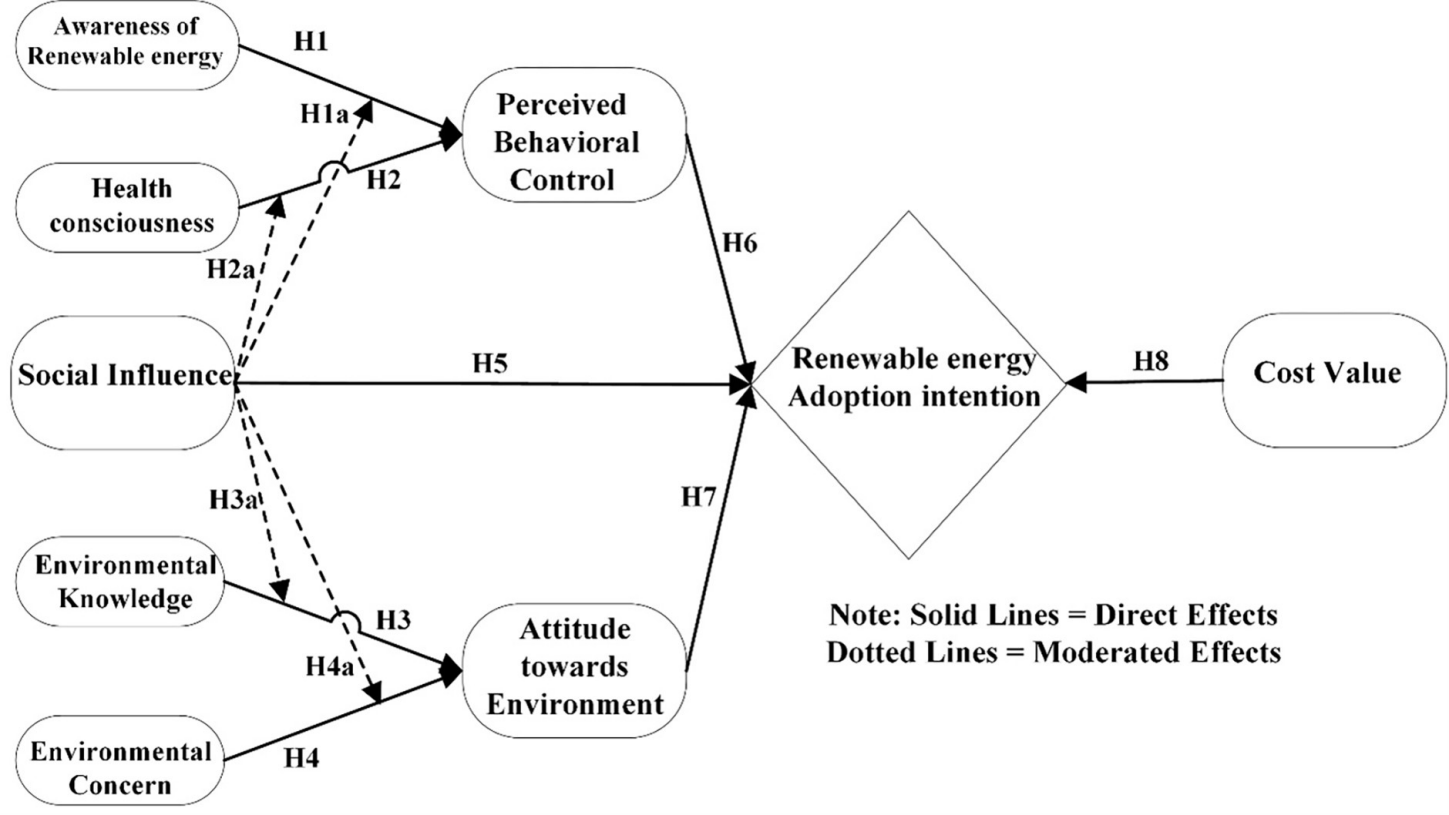
Conceptual framework.

According to Ajzen’s 1991 [15] cognitive theory of planned behaviour (TPB), an individual’s decision to engage in a given action may be influenced by their level of motivation to carry it out. To paraphrase, "Intentions are considered to reflect the motivating variables that impact behaviour; they are signs of how hard individuals are willing to try, how much of an effort they intend to expend in order to do the behaviour." The greater one’s purpose in undertaking a behaviour, the greater one’s chance of actually doing it [15]. Our integrated suggested model (Fig 1) is based on this line of thinking about the mind. We hypothesise that a household’s behaviour in renewable energy adoption will be influenced by perceived behavioural control, attitude towards the environment, cost value, and social influence. We have also hypothesised that PBC is a mediator between HC, ARE, and renewable energy adoption, and ATE is a mediator between EK, EC, and renewable energy adoption intentions. We further assume that these relationships are moderated by social influence.

### Hypothesis development

#### Health Consciousness (HC)

Health consciousness refers to the level of concern people have for their physical and mental well-being. Individuals are more likely to adopt preventative health care measures if they already engage in health-promoting behaviours, which in turn increases the likelihood that people will be health-conscious [16]. If human being is concerned about their health and the environment, they will use eco-friendly products that help improve their health and overall ecological system [17]. Researchers have discovered that health consciousness significantly influences the behavioural intention to buy new technology [18]. They further argue that health-conscious people are more inclined toward the technologies and products that are good for health and the environment. Study in the past has also shown the importance of two psychological concepts in predicting health-related behaviours: sensitivity to others’ feelings and health consciousness [19]. The brand’s perceived worth and the consumers’ attitudes about it are highly impacted by the consumers’ health awareness and social status. Furthermore, perceived significance, brand loyalty, and brand attitude all have direct effects on the propensity to buy [20]. Health consciousness shape humans perceived behaviour control that leads to the intention of adoption or rejection of a particular technology, service, or product. Researchers in the past have also proved that social influence substantially impacts an individual’s choice about the use of services connected to technology [21, 22]. When compared to customers who place a lower importance on protecting their personal information, consumers who place higher importance on protecting their personal information have weaker connections between their perceived level of comfort and their desire to embrace new technologies [13]. Social influence acts as a catalyst in the adoption or rejection of new technology purchases; we assume that if social influence is higher, people will be less caring about their health and environment in developing countries where health-related awareness is less and people are cost sensitive. Hence we have hypothesised the following.

H1: Health consciousness influences perceived behaviour control.

H1a: Perceived behavioural control mediates the relationship between health consciousness and renewable energy adoption.

H1b: A higher level of Social influence weakens the role of perceived behavioural control between Health consciousness and Renewable energy adoption for more socially influenced households.

#### Awareness of Renewable Energy (ARE)

A person’s adoption or rejection of a technical advance is based on their level of awareness, which may be defined as their comprehension or recognition of the benefits and disadvantages of the invention, product, or service [18]. This component of consumers’ desire to embrace renewable energy plants has received less attention from researchers. Researchers have shown that many people are unaware of the advantages of using renewable energy plants in the home, especially solar panels, to produce electricity [23]. This lack of information hinders households’ consideration of renewable energy plants. Similar to the findings of other studies, it has been observed that e-commerce adoption is strongly influenced by people’s knowledge of possible threats and opportunities [24]. When discussing renewable energy for the home, we’re presuming that people are as interested in installing them as they are knowledgeable about them. Researchers have also revealed that awareness of renewable energy is a significant predictor of its use [25] among Korean and Indian students. Aware households are more inclined toward adopting cost-effective and environmentally friendly products in developing countries than less aware households [17]. When people interact and socially interchange their issues and information, they gain knowledge about different products and technologies that are good for society and a community member’s personal interest [26]. We assume that socially influenced community members will have more awareness compared to the household who do not prefer to take part in social interactions actively; hence we hypothesise that

H2: Awareness of renewable energy influences perceived behaviour control.

H2a: Perceived behavioural control mediates the relationship between ARE and renewable energy adoption.

H2b: More socially influenced households are more aware of renewable energy, and the role of perceived behavioural control between ARE and Renewable energy adoption is stronger as social influence increases.

#### Environmental Concern (EC)

Concern for the environment may be defined as an individual’s awareness of the fact that the actions of both themselves and others have an impact on the surrounding environment [27]. Or, in other words, the conviction that the actions of oneself and others might have a negative impact on the environment [28]. A person who has acquired anxiety about the atmosphere has a high tendency to adopt or participate in behaviours that are ecologically beneficial. According to researchers [29], a person’s care for the environment may be used to predict their attitude toward the environment if they buy environmentally friendly items. In addition, researchers in the past said that consumers’ environmental concern affects responsible consumption by changing people’s attitudes about the environment [30]. Being concerned about the environment makes it more likely that one will choose the option that has a less negative influence on the natural world rather than the more damaging one. This process is mediated by the perceived social danger connected with the ecologically unfriendly product and the self-incongruity that this risk creates [31]. Consumers concerned about the environment are more likely to purchase green products [32]. According to the research, individuals who have a gloomy outlook do not often purchase organic food. On the other hand, they have a deep concern for the environment, which, as a result, is likely to transform them from pessimistic customers into optimistic buyers who start to purchase organic foods. In addition, consumers who are gloomy about the future are considerably motivated by social influence to embrace organic goods, which helps to minimise the concern-behaviour gap [33]. It seems that modifying environmental ideals and encouraging environmentally conscious conduct may be complemented by learning about sustainable behaviour from one’s peers. The results demonstrate that the promotion of environmentally friendly consumption habits has obvious local externalities that surpass the global implications. This means that the capacity to learn in small networks is essential for the development of trust and the interchange of ideas and practises [34, 35]. With this literature debate, we have hypothesised the following.

H3: Environmental concerns influence the attitude toward the environment.

H3a: Attitude towards the environment mediates the relationship between EC and renewable energy adoption.

H3b: Social influence strengthens the mediation role of attitude towards the environment between EC and Renewable energy adoption.

#### Environmental Knowledge (EK)

Individuals’ knowledge and comprehension levels may be inferred from the behaviours and acts they engage in. People may better understand environmental concerns and how to behave ecologically responsibly if they have eco-literacy. Customers that are environmentally concerned have a greater inclination to buy environmentally friendly items [32]. Knowledge of the environment is positively connected with people doing activities that are beneficial to the environment [17]. People who have a greater comprehension of a topic and respond to it in a manner that is more positive and constructive are considered to have a higher level of engagement than those who have a lesser understanding of the topic. Environmental intelligence has the capacity to impact how consumers perceive the environment immediately around them, which in turn has the power to change consumers’ intentions towards the behaviour they intend to engage in. According to several research findings, consumers’ environmental knowledge and preferences directly impact their products [17, 32, 36]. The extent to which a person is eco-literate may influence their actions. Furthermore, it has been shown that environmental information positively influences people’s perceptions of environmental risk, environmental issues, and green buying habits. In a recent study conducted in one of the developing nations, researchers found that customer interest in eco-friendly items was highly influenced by environmental literacy [17]. It has prompted people to switch to greener technology like 5G [18]. So, we think people will want to switch to green energy sources like wind turbines, solar panels, and biogas plants if they are informed about the environment and the financial and environmental costs associated with conventional energy sources like coal and natural gas. It is with this in mind that we put forth.

H4: Environmental knowledge influence the attitude toward the environment.

H4a: Attitude towards the environment mediates the relationship between EK and renewable energy adoption.

H4b: Social influence strengthens the mediation role of attitude towards the environment between EK and Renewable energy adoption.

#### Cost Value (CV)

Consumers are influenced by several economic factors while making purchases. Customers’ purchasing choices are recognised to be highly impacted by their financial situations [18, 37, 38]. As per researchers, the word "cost value" indicates the benefit that is seen to be provided by a product, less the costs that are involved with the purchase of such items [37]. There isn’t a single academic discipline that doesn’t influence economic issues. Economic considerations play a significant influence when it comes to the acceptance or rejection of new technology. People have the propensity to accept or reject technology that falls within their financial means. If we are discussing the implementation of new technologies, economic aspects would include cost value and circumstances that facilitate change. The notion of having both cheap expenses and the possibility of a return on investment is what we mean when we talk about having excellent value. Users of a certain technology tend to base their decision on what was supplied against what was taken away when they consider the advantages and expenses of using the technology. Consumers’ willingness to accept new technologies was most strongly correlated to the price at which such technologies were offered [32]. When making a purchase, individuals often factor in pricing information in order to deal with financial shortfalls [39]. Renewable energy installation is costly because of the high initial investment required for new technological ventures. Renewable energy inflated price tags put customers off. Another study indicated that the price put many people off in renewable energy use [40]. The research was conducted to learn what variables in the United Arab Emirates’ retail sector are driving the adoption of green energy technology adoption. The research showed that the economy and the environment are the two most important factors influencing consumer opinion in the United Arab Emirates [41]. With this, we have hypothesised the following.

H5: Cost value influences renewable energy adoption.

#### Social Influence (SI)

We say that two people are "connected" to one another in a social network if and only if there is some link or "tie" [42]. Individuals, groups, states, or nations may all play a role as actors. The term "alters" is used to refer to the members of a group that are linked to the actors. Researchers have looked at how social networks might impact behaviour and have found that the effects can be broken down into two primary mechanisms: information dissemination and social influence. The first mechanism is the spread of knowledge about technology across a community, which may lessen the need for training and education as well as the associated risks associated with adopting it. Researchers claim that this phenomenon is more common when an individual seeks out knowledge and builds beliefs from members of the network who have a specific status and accurate knowledge [7].

The second is what psychologists call "social influence," or the desire to conform to the norms of one’s peers in order to "fit in," either to avoid confrontation or to seem to have the approval of one’s peers. The formation of social standards that individuals follow to prevent conflicts with other members of their communities or as a sort of obedience is one example of how people are influenced socially [7]. In cases when the act of adoption includes an extra meaning, such as conveying agreement or support for a shared goal, this phenomenon is often called the endorsement effect. Adoption rates and other aggregate statistics on the behaviour of network members are used in this process to guide adoption decisions. Researchers [43] proposed utilising observational data to separate the impacts of information dissemination and social influence as an alternate method. In particular, the author demonstrates that once beliefs are included, network effects indicate strategic conduct, either in the form of strategic delay or social pressure. According to research, family and friends, trustworthy network members, and people from outside the community all significantly influence the correlation between information dissemination and biogas adoption. On the other hand, there is no noticeable link between official government data and the uptake of biogas [7]. Based on this prevailing literature, we have hypothesised a direct relationship between social influence with renewable energy adoption and its moderated role in our model (Fig 1).

H6: Social influence affects renewable energy adoption.

#### Attitude towards Environment (ATE)

An individual’s attitude may be defined as the evaluative posture they adopt toward the behaviour being discussed. An individual’s perspective on the environment may be defined as their assessment of the degree to which they believe the environment to be significant [44]. In addition, a person’s behaviour demonstrates a behavioural attitude toward the necessity of conserving the environment, and these acts are verified by suitable activities [15]. In addition, attitude is a significant factor in determining an individual’s desire to engage in certain behaviours [15, 45]. Beliefs about behaviour and evaluations of consequences have a role in the formation of attitudes toward the natural world. Beliefs that the natural world is essential and that it is essential to safeguard the natural world are particularly influential [46]. We assume that an individual’s environmental knowledge and EC will shape their attitude towards the environment, and it will be a bridge between the environmental knowledge, environmental concern, and renewable energy adoption of households. Hence we have hypothesised the following.

H7: Attitude towards the environment affect renewable energy adoption.

#### Perceived Behavioural Control (PBC)

One’s sense of how simple or complex it is to carry out a certain behaviour is known as "perceived behavioural control" [15]. TPB states that "intention is the best predictor of behaviour, and that intention depends on three factors: attitude towards a behaviour, subjective norms, and the individual’s perceived behavioural control" [15]. Thus, according to the theory, those with more favourable views, subjective standards, and perceived behavioural control are more inclined to act on those intentions and carry them through [15]. Despite favourable attitudes and supportive subjective standards, people’s intentions to act may not change if they feel they lack sufficient control over their actions. It has been argued that bridging the gap between consumers’ positive attitudes toward sustainable practices and their actual purchase behaviour requires empowering consumers by giving them greater control over their behaviours. This would be accomplished by increasing the consumers’ perceived level of behavioural control. Evidence reveals that customers are more likely to act in a desirable manner when they feel they have a great deal of influence over their decisions to buy organic food goods [47]. The researchers have already revealed a positive correlation between perceived behavioural control and future behaviour [48]. Hence we have hypothesised the following.

H8: Perceived behavioural control affect renewable energy adoption.

## Methods

### Data collection

**Fig 2:**
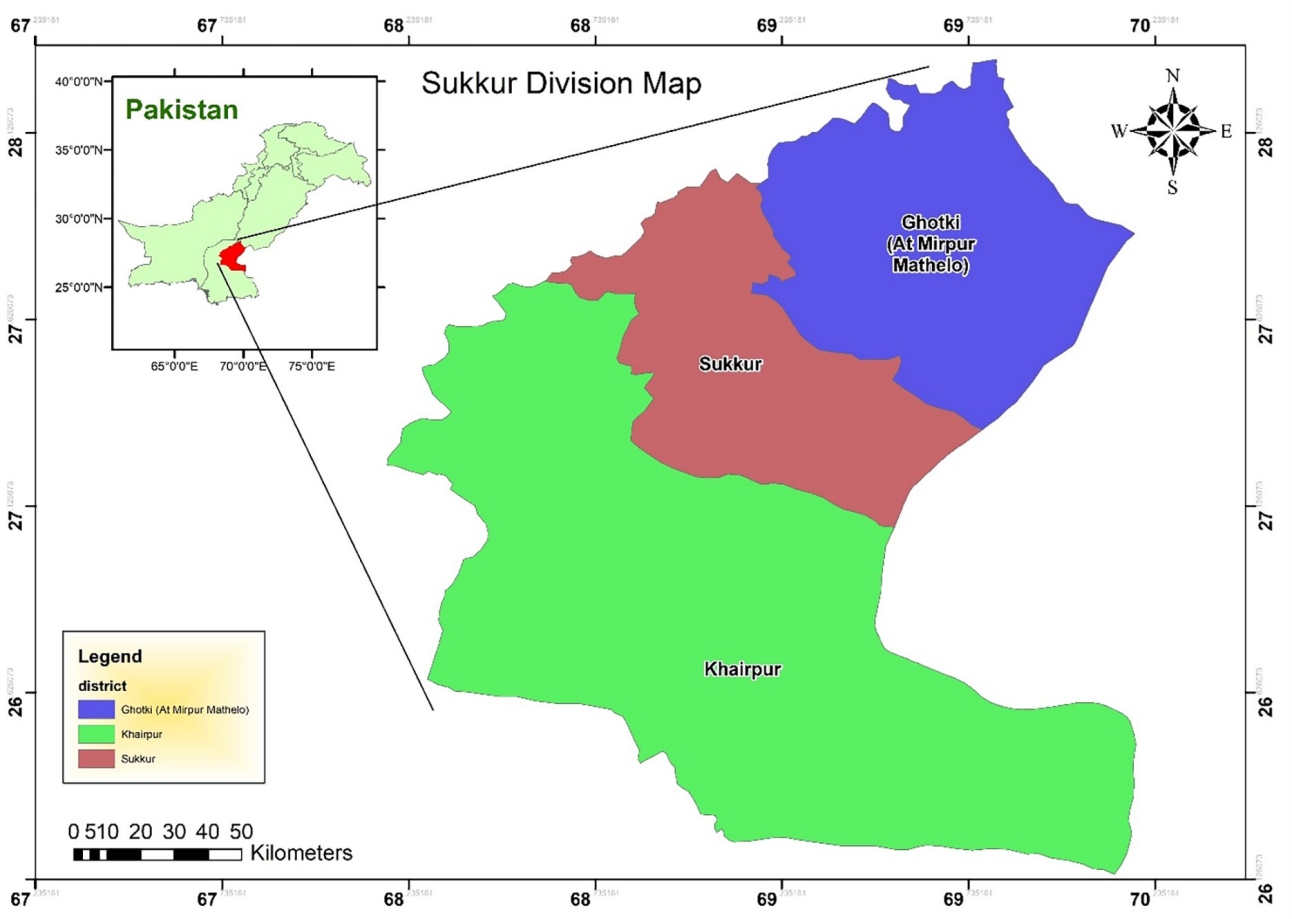
Study area.

In this research, a survey was employed as the data collection technique, and questionnaires were distributed to households in the city of Sukkur, situated in the province of Sindh in Pakistan (Fig 2). It is the second biggest city in the province of Sindh, with a population of 0.577 million. The reason behind the selection of this particular city is its identification as one of the high-potential wind energy areas [49].

In order to acquire the dataset, we relied on a validated construct derived from earlier research. The supplementary material presents the detailed measurement items of the construct utilised to obtain the sample response. The measuring items’ phrasing has been altered very little so that they are more compatible with our research and that we may more properly gather responses. Before starting a formal data collection, we performed a pilot study to ensure the consumption time of respondents’ understanding and language difficulties. We have picked 20 random students of master’s level from a local university for the pilot study [50]. The pilot study results were favourable and encouraged us to continue the study. Because of the potential for bias, the respondents from the pilot research were excluded from the final sample.

We have adopted a random sampling technique based on the electricity billing data and picked every 50^th^ household. It is a good approach to collecting samples from a heterogeneous population [51]. A total of 1200 questionnaires were distributed, and we received 840 complete responses for statistical analysis with a response rate of 70%. The survey was carried out for one month from the third week of April 2022.

Before collecting any information or responses from any of the respondents, the researchers made sure to explain the goal of the study to each one of them and get their agreement. For the purpose of measuring the reaction, we have used a Likert scale of seven points, with "1 indicating strongly disagreeing and 7 as strongly agreeing." According to the findings of the aforementioned research, the Likert scale with seven points is superior to higher-order alternative scales since it is both more accurate and simpler to use [52, 53]. Authors have received approval from the Beijing University of Technology’s Ethics committee to conduct this study. In addition, all participants who participated in this study were informed about the purpose of data collection, and the questionnaire was shared after the consent of the participants. Every questionnaire started with a purpose statement explaining the collected dataset’s use. All authors and the ethics committee approved the study protocols. No minor was involved in this study.

### Demographics of respondents

We have compiled the respondent’s age, education level, gender, employment, residence status, and other relevant information to have a more comprehensive understanding of our research sample and its features. Table 1 provides an in-depth look at the demographic information of our whole sample size (840), which may be seen below.

**Table 1:** Demographic characteristics.

* Characteristics*	*Range*	*Frequency*	*Percentage*
Gender	Male	453	53.9%
	Female	387	46.1%
Education level	High School or Less	124	14.8%
	Bachelor	319	38.0%
	Master	371	44.2%
	Doctorate	26	3.1%
Age	18-25Year	205	24.4%
	26-35Year	275	32.7%
	36-45Year	268	31.9%
	>45Year	92	11.0%
Residential status	Rural	312	37.1%
	Urban	528	62.9%
Occupation	Student	219	26.1%
	Govt. Employee	174	20.7%
	Private Company Employee	266	31.7%
	Businessman/women/other	181	21.5%

### Common method variance

A significant issue that may arise in a survey sample is known as common method variance (CMV) bias. In the current investigation, "Harman’s single-factor test" was used in order to determine the CMV [54]. In order to determine whether or not CMV is present among the constructs, a test based on a single factor was devised [55, 56]. The findings demonstrated that all the sample items could be broken down into 9 distinct factors, with the first component responsible for 42.2% of the total variance, which is lower than the recommended criterion of 50% (S1 File—contains all the supporting tables). In addition, we carried out a comprehensive collinearity analysis by making use of a technique known as Smart partial least squares (Smart-PLS). According to many academics in the social sciences, this approach is relatively effective and exact [36, 57, 58]. Because not a single VIF value is higher than the recommended threshold of 5, it can be concluded that the common problem of process bias is not present in our model [37].

## Results

For the purpose of analysing the results of the survey in this investigation, the Smart PLS 4.0 software package was used [59]. The PLS-SEM analysis is carried out in two stages: first, the measurement model is analysed, and then the structural model is examined [59, 60]. Only constructs that have acceptable indicator loading, composite reliability, convergent validity, and discriminant validity will be used in the structural model since it is a requirement of the measurement model. Using the bootstrapping method, the structural model evaluation determines the route coefficients’ size after evaluating them. The approach proposed by [61] was chosen for the moderated mediation study because it is the method that ensures the most accurate testing of moderated mediating effects and is better suitable for using the PLS-SEM method [59, 61].

### Measurement model assessment

**Fig 3:**
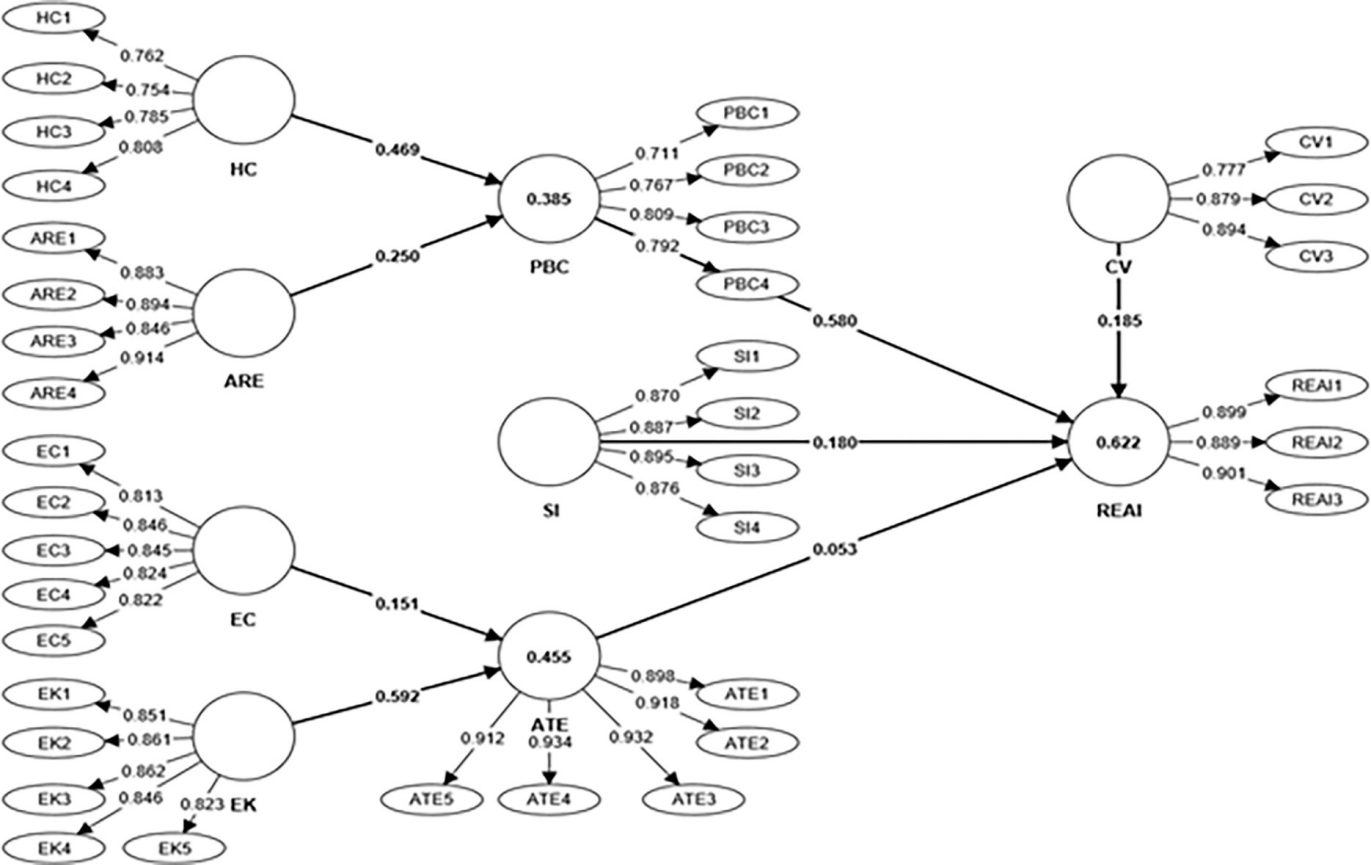
Measurement model.

Outer loading for our model falls in the range of 0.711 to 0.934, and all indicators have a degree of reliability that is sufficient to be considered reliable (Table 2, Fig 3). The VIF values presented in Table 2 also indicate that the multicollinearity issue does not exist in the study dataset, as the values of VIF are far less than the recommended threshold level of 5. The results of the measurement reliability are shown in Table 3, which reveals Cronbach’s alpha, rho_A, and composite reliability scores are under the prescribed threshold level [59]. In addition, the values of AVE range from 0.605 to 0.845, which shows that variables are converging toward a single value. Consequently, the findings demonstrate that all reflective measuring methods can meet the essential assessment criteria. The concept of discriminant validity refers to the ways in which one construct statistically varies from another construct. We have utilised two renowned criteria to assess the discriminant validity, Fornell and Larcker criteria and heterotraitmonotrait ratio [62]. Results presented in Table 4 revealed that the square root of AVE (diagonal values) is the highest in correlation to the relative values. Hence it fulfils the F&L criteria for discriminant validity [63, 64]. Researchers argue that HTMT is a workable technique when there is just a small difference in loading. When two LVs have the same notion, the HTMT is 0.90, but when they have different ideas, it is just 0.85. According to Table 5, all of the LVs have values lower than 0.85 in the first order, demonstrating that the discriminant validity criteria have been satisfied [62].

**Table 2:** Construct reliability and validity.

*Constructs*	*Items*	*Loadings*	*VIF*	*STDEV*	*T-Statistics*
Awareness of Renewable energy	ARE1	0.883***	2.603	0.009	94.483
	ARE2	0.894***	2.888	0.008	113.382
	ARE3	0.846***	2.32	0.012	68.177
	ARE4	0.914***	2.438	0.008	120.775
Attitude towards environment	ATE1	0.898***	2.187	0.008	116.035
	ATE2	0.918***	2.979	0.006	149.348
	ATE3	0.932***	2.134	0.007	140.553
	ATE4	0.934***	2.847	0.005	198.164
	ATE5	0.912***	2.332	0.005	169.242
Cost Value	CV1	0.777***	1.499	0.023	34.155
	CV2	0.879***	2.068	0.011	79.076
	CV3	0.894***	2.14	0.01	86.348
Environmental concern	EC1	0.813***	2.814	0.014	57.798
	EC2	0.846***	2.066	0.011	74.102
	EC3	0.845***	2.762	0.013	67.491
	EC4	0.824***	2.394	0.014	60.121
	EC5	0.822***	2.608	0.015	56.292
Environmental Knowledge	EK1	0.851***	2.702	0.01	89.525
	EK2	0.861***	2.914	0.009	92.574
	EK3	0.862***	2.765	0.009	92.473
	EK4	0.846***	2.859	0.012	71.148
	EK5	0.823***	2.559	0.012	70.111
Health Consciousness	HC1	0.762***	1.621	0.02	38.641
	HC2	0.754***	1.577	0.018	42.953
	HC3	0.785***	1.556	0.017	45.044
	HC4	0.808***	1.622	0.015	55.532
Perceived Behavioural Control	PBC1	0.711***	1.399	0.026	27.711
	PBC2	0.767***	1.518	0.019	40.712
	PBC3	0.809***	1.643	0.015	53.080
	PBC4	0.792***	1.539	0.013	59.683
Renewable energy adoption intention	REAI1	0.899***	2.328	0.008	117.582
	REAI2	0.889***	2.382	0.009	95.912
	REAI3	0.901***	2.515	0.009	103.217
Social Influence	SI1	0.870***	2.393	0.009	93.859
	SI2	0.887***	2.705	0.011	83.053
	SI3	0.895***	2.83	0.008	110.229
	SI4	0.876***	2.688	0.009	96.743

Notes: VIF<5; ***Significant at p < 0.*001*

**Table 3:** Construct reliability and validity.

*Variables*	*Mean*	*STDEV*	*Skewness*	*Cronbach’s alpha*	*rho_A*	*CR*	*AVE*
ARE	5.1425	1.401	-1.127	0.907	0.911	0.935	0.783
ATE	3.689	1.175	0.662	0.954	0.954	0.965	0.845
CV	4.9091	1.359	-0.699	0.81	0.827	0.888	0.725
EC	3.3476	0.656	0.113	0.888	0.893	0.917	0.689
EK	3.0605	0.712	0.31	0.903	0.907	0.928	0.72
HC	4.5232	1.337	-0.422	0.783	0.79	0.86	0.605
PBC	4.6399	1.393	-0.468	0.772	0.779	0.853	0.593
REAI	4.9258	1.429	-0.799	0.878	0.88	0.925	0.803
SI	5.3092	1.208	-1.046	0.905	0.907	0.933	0.778

Notes: *α >0*.*7; CR > 0*.*7; AVE > 0*.*5*.

ARE: Awareness of Renewable energy, ATE: Attitude towards environment, CV: Cost Value, EC

Environmental concern, EK: Environmental Knowledge, HC: Health Consciousness, PBC: Perceived Behavioural Control, REAI: Renewable energy adoption intention, SI: Social Influence

**Table 4:** Discriminant validity (Fornell-Larcker criterion).

	*ARE*	*ATE*	*CV*	*EC*	*EK*	*HC*	*PBC*	*REAI*	*SI*
ARE	**0.885**								
ATE	-0.043	**0.919**							
CV	0.713	-0.022	**0.852**						
EC	-0.04	0.422	-0.033	**0.83**					
EK	-0.035	0.661	0.023	0.457	**0.849**				
HC	0.433	0.025	0.467	-0.026	0.016	**0.778**			
PBC	0.453	-0.041	0.447	0.031	-0.044	0.578	**0.77**		
REAI	0.575	0.018	0.558	0.022	0.038	0.697	0.731	**0.896**	
SI	0.713	-0.036	0.641	0.009	-0.016	0.464	0.396	0.525	**0.882**

**Table 5:** Discriminant validity (Heterotrait-Monotrait ratios).

	*ARE*	*ATE*	*CV*	*EC*	*EK*	*HC*	*PBC*	*REAI*	*SI*
ARE									
ATE	0.061								
CV	0.823	0.032							
EC	0.051	0.454	0.049						
EK	0.068	0.708	0.07	0.507					
HC	0.505	0.066	0.577	0.081	0.059				
PBC	0.54	0.055	0.564	0.07	0.053	0.736			
REAI	0.64	0.021	0.655	0.053	0.057	0.831	0.877		
SI	0.783	0.043	0.744	0.037	0.057	0.542	0.471	0.587	

ARE: Awareness of Renewable energy, ATE: Attitude towards environment, CV: Cost Value, EC

Environmental concern, EK: Environmental Knowledge, HC: Health Consciousness, PBC: Perceived Behavioural Control, REAI: Renewable energy adoption intention, SI: Social Influence

In addition to these two criteria, the cross-loading table is also presented as supplementary material.

### Structural model

**Fig 4:**
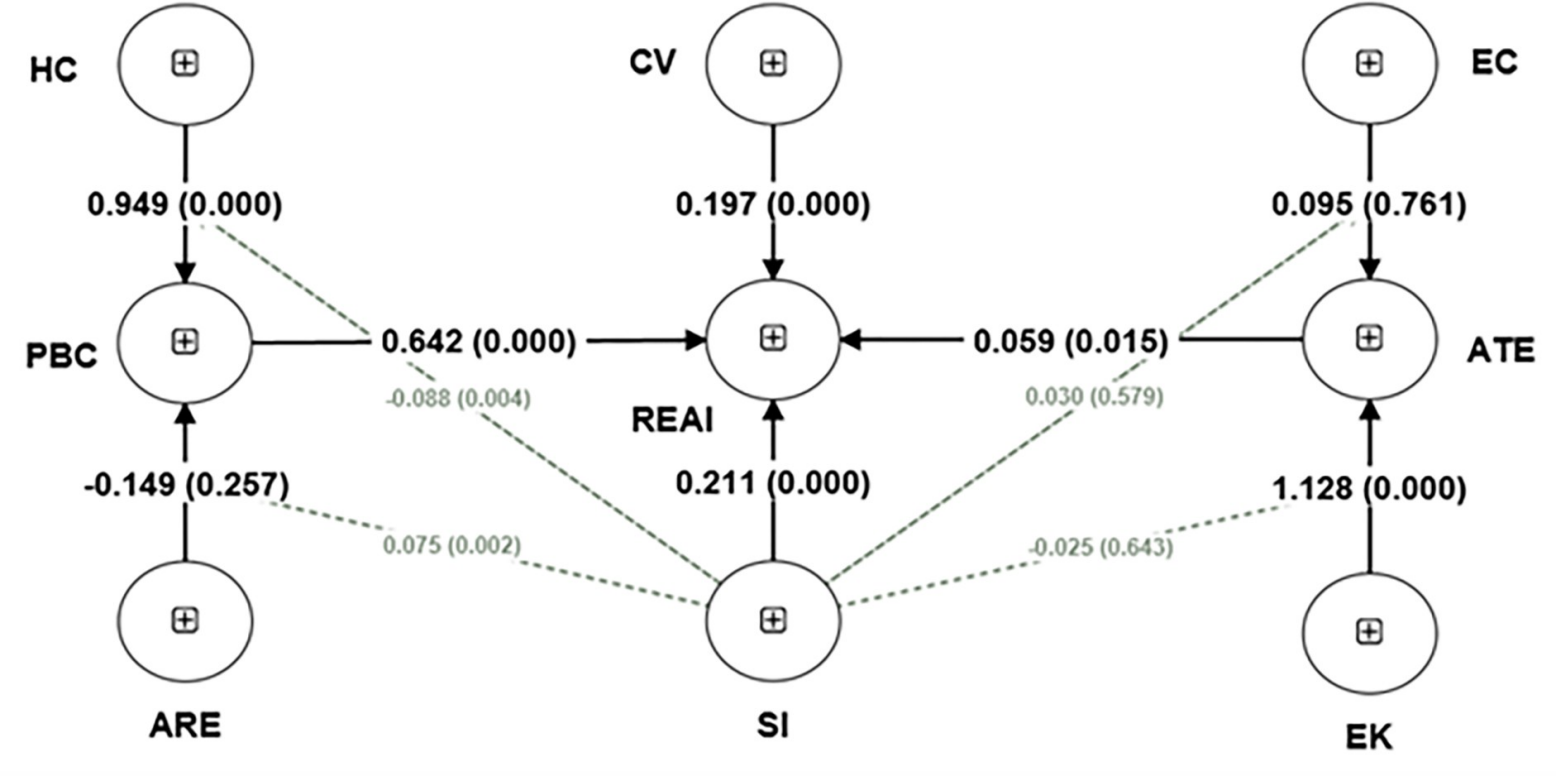
Path model.

After confirming the construct’s reliability and validity, the next step in PLS-SEM is to run a path model to test the direct and indirect relationships proposed in the model and the model’s quality and accuracy in predicting the results. We have followed the recommended procedure of earlier researchers for direct relationships and conditional mediation [59, 61]. We have used the recommended 5000 subsampling during bootstrapping. In previous versions of Smart-PLS software, researchers needed to perform several steps and run different models to access the moderated meditation, but in the new version of Smart-PLS 4.0, it can be analysed in a single model. Fig 4 depicts the results of our path model with beta and p-values in parenthesis. Table 6 presents the quality criteria of our model; it can be seen that R^2^ and adjusted R^2^ values present the high prediction power of our model. Exogenous constructs explained (Adjusted R^2^) ATE = 44.8%, PBC = 38.8%, and REAI = 60.8% variance.

**Table 6:** Quality criteria.

***R*** ^***2***^					***Confidence Interval***	
	Original sample (O)	Standard deviation (STDEV)	T statistics	P values	2.50%	97.50%
ATE	0.452	0.03	15.14	0.000	0.397	0.513
PBC	0.392	0.03	12.872	0.000	0.333	0.455
REAI	0.61	0.025	24.779	0.000	0.562	0.658
***R***^***2***^ ***Adjusted***					***Confidence Interval***	
	Original sample (O)	Standard deviation (STDEV)	T statistics	P values	2.50%	97.50%
ATE	0.448	0.03	14.941	0.000	0.393	0.51
PBC	0.388	0.031	12.677	0.000	0.329	0.451
REAI	0.608	0.025	24.585	0.000	0.56	0.657

Further, we have used t-statistics and p-value to determine the significance of path coefficients (Table 7). Results presented in Table 7 demonstrate that HC->PBC (β = 0.949; p-value<0.001), CV->REAI (β = 0.197; p-value<0.001), SI->REAI (β = 0.211; p-value<0.001), EK->ATE (β = 1.128; p-value<0.001), PBC->REAI (β = 0.642; p-value<0.001), ATE->REAI (β = 0.059; p-value = 0.015) are significant but ARE->PBC (β = -0.149; p-value = 0.257), EC->ATE (β = 0.095; p-value = 0.761) are insignificant. Hence H1, H4, H5, H6, H7, and H8 are supported, while H2 and H3 are rejected.

**Table 7:** Path model results.

*Direct Relationship*						
					Confidence Interval	
*Paths*	*β*	*STDEV*	*T statistics*		*2*.*50%*	*97*.*50%*
ARE -> PBC	-0.149^NS^	0.132	1.134		-0.409	0.104
ATE -> REAI	0.059***	0.024	2.43		0.011	0.105
CV -> REAI	0.197***	0.038	5.244		0.123	0.27
EC -> ATE	0.095^NS^	0.311	0.304		-0.509	0.701
EK -> ATE	1.128***	0.282	4.004		0.586	1.688
HC -> PBC	0.949***	0.169	5.605		0.622	1.285
PBC -> REAI	0.642***	0.035	18.251		0.573	0.711
SI -> REAI	0.211***	0.035	6.075		0.144	0.279
SI x EC -> ATE	0.03^NS^	0.054	0.555		0.579	-0.074
SI x EK -> ATE	-0.025^NS^	0.053	0.463		0.643	-0.131
SI x ARE -> PBC	0.075***	0.024	3.111		0.002	0.028
SI x HC -> PBC	-0.088***	0.03	2.92		0.004	-0.148
**Indirect Relationships**						
HC -> PBC -> REAI	0.609***	0.112		5.441	0.396	0.839
SI x HC -> PBC -> REAI	-0.057***	0.019		2.948	-0.095	-0.019
SI x EC -> ATE -> REAI	0.002^NS^	0.003		0.517	-0.005	0.009
SI x EK -> ATE -> REAI	-0.001^NS^	0.003		0.44	-0.008	0.006
EK -> ATE -> REAI	0.066**	0.03		2.186	0.011	0.13
ARE -> PBC -> REAI	-0.096^NS^	0.085		1.128	-0.266	0.067
SI x ARE -> PBC -> REAI	0.048***	0.016		3.031	0.018	0.08
EC -> ATE -> REAI	0.006^NS^	0.02		0.278	-0.031	0.05

Note

***Significant at p < 0.001

**Significant at p<0.05.

**Fig 5:**
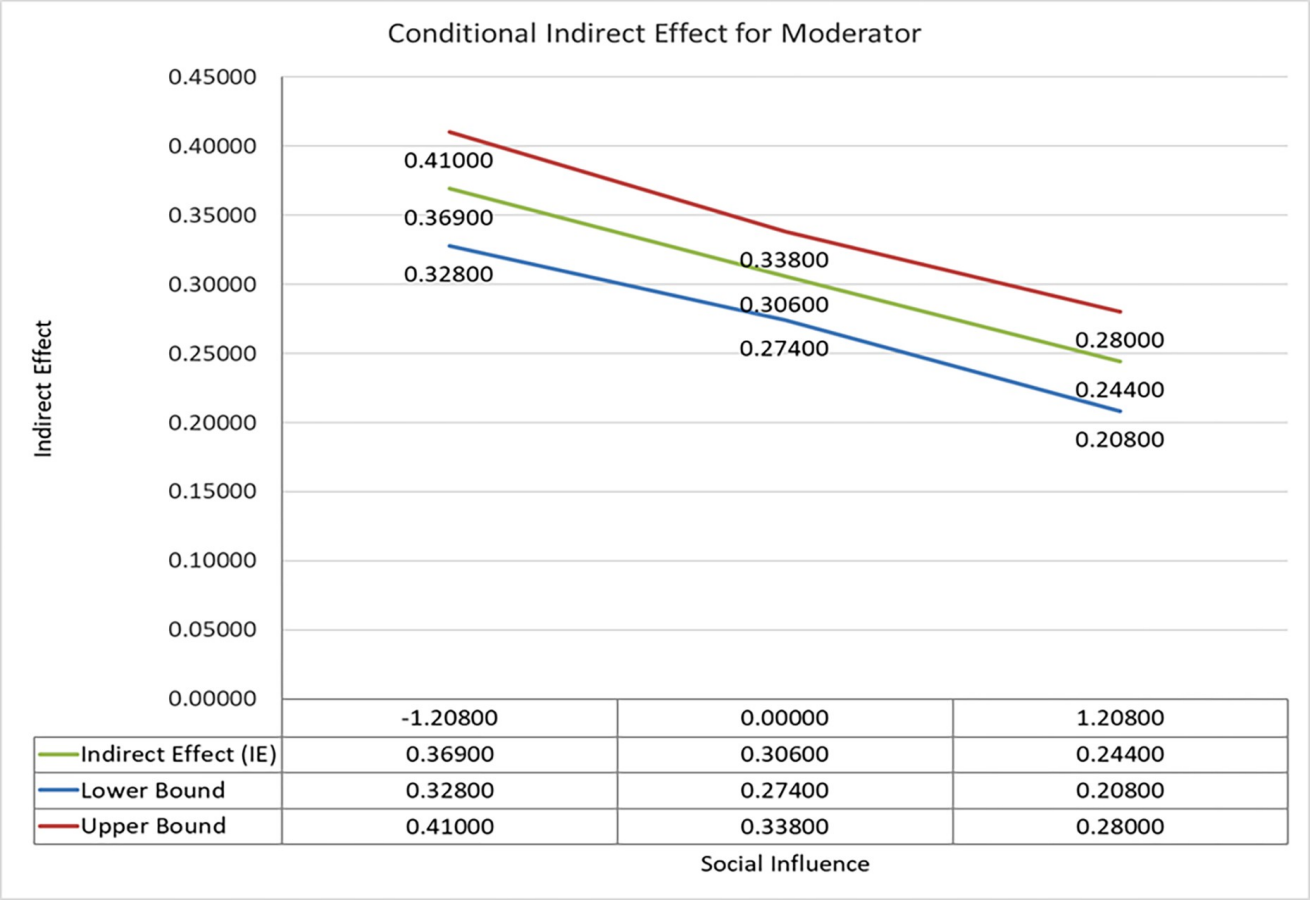
A plot of conditional moderation effect (SI x HC -> PBC -> REAI).

**Fig 6:**
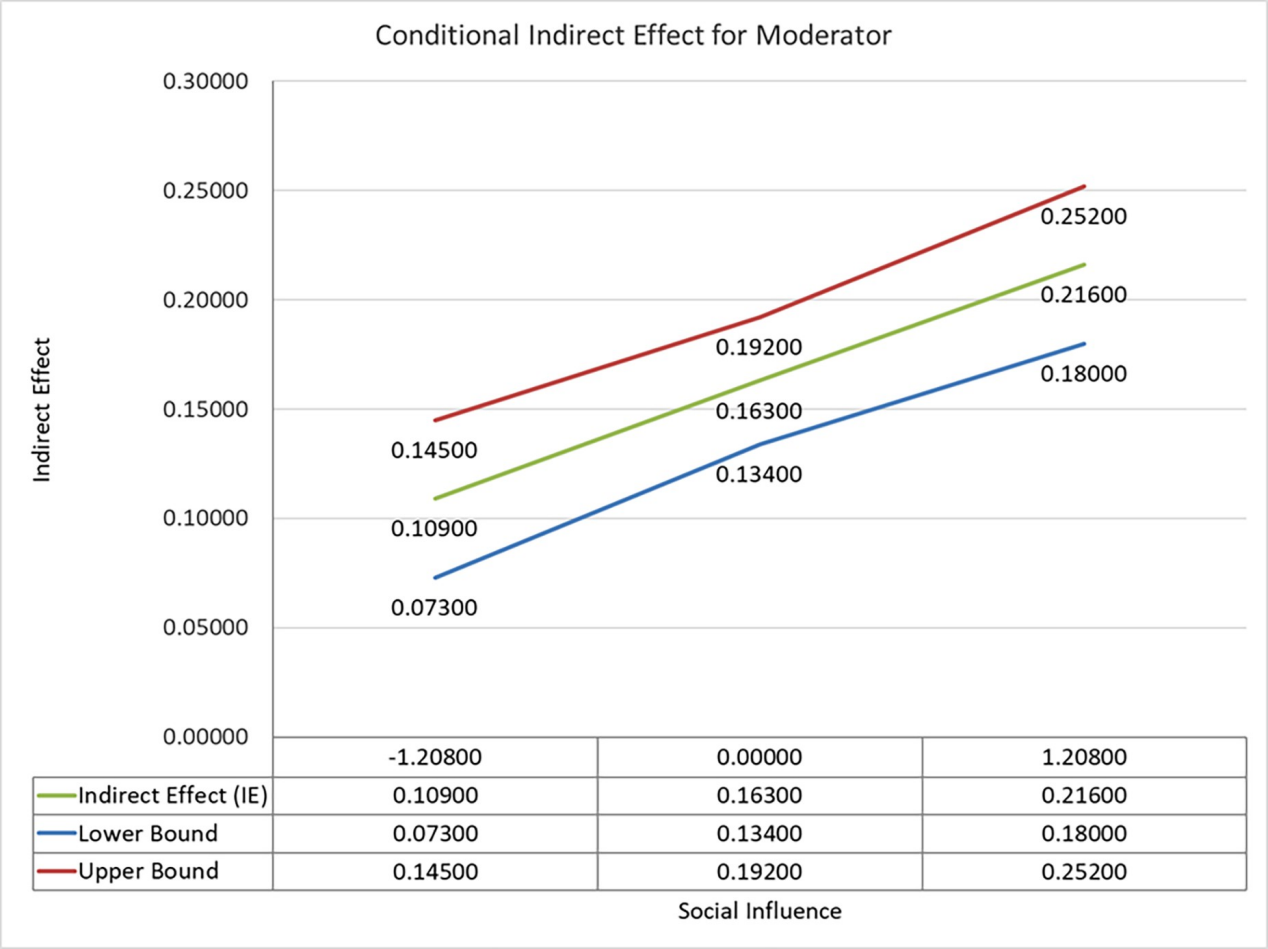
A plot of conditional moderation effect (SI x ARE -> PBC -> REAI).

Moderated indirect effects for low, high and mean levels of social influence are presented in Table 8. The results indicate that conditional mediation is significant for all levels of the proposed model. We have presented all the specific mediation results in Table 8 but draw plots (Figs 5 and 6) for specific significant indirect relationships presented in Table 7, i.e., SI x HC -> PBC -> REAI (β = -0.057: t-value = 2.948) and SI x ARE -> PBC -> REAI(β = -0.048: t-value = 3.031).

**Table 8:** Moderated indirect effect.

*Conditional Indirect Effect of Moderator*				*Confidence Interval*	
Paths	β	STDEV	T statistics	2.50%	97.50%
ARE -> PBC -> REAI conditional on SI at +1 SD	0.216***	0.036	5.965	0.148	0.291
EC -> ATE -> REAI conditional on SI at +1 SD	0.017***	0.008	2.214	0.003	0.033
EK -> ATE -> REAI conditional on SI at +1 SD	0.057***	0.025	2.278	0.01	0.109
HC -> PBC -> REAI conditional on SI at +1 SD	0.244***	0.036	6.842	0.176	0.316
ARE -> PBC -> REAI conditional on SI at -1 SD	0.109***	0.031	3.535	0.051	0.17
EC -> ATE -> REAI conditional on SI at -1 SD	0.013^NS^	0.008	1.596	0	0.032
EK -> ATE -> REAI conditional on SI at -1 SD	0.06***	0.025	2.407	0.011	0.109
HC -> PBC -> REAI conditional on SI at -1 SD	0.369***	0.041	9.061	0.293	0.453
ARE -> PBC -> REAI conditional on SI at Mean	0.163***	0.029	5.671	0.108	0.22
EC -> ATE -> REAI conditional on SI at Mean	0.015***	0.007	2.154	0.002	0.03
EK -> ATE -> REAI conditional on SI at Mean	0.059***	0.025	2.368	0.01	0.107
HC -> PBC -> REAI conditional on SI at Mean	0.306***	0.032	9.623	0.246	0.371

Note

***Significant at p < 0.001

**Significant at p<0.05.

We followed the earlier researcher’s instructions and plotted the moderator’s conditional indirect effect [61] to better understand and clarify the relationship. The plot for path SI x HC -> PBC -> REAI (Fig 5) reflects that social influence weakens the relationship mediation effect (β = -0.057: t value = 2.948). It means as social influence increases, the effect of HC on REAI via PBC decreases significantly and vice versa.

Results presented in Fig 6 for the specific path SI x ARE -> PBC -> REAI (β = 0.048; t-value = 3.031) reflects that with a high level of social influence, the effect of ARE on REAI through perceived behavioural control increases, i.e., it has a direct positive effect as social influence increases the effect of awareness of renewable energy on renewable energy adoption intentions increases. The plot (Fig 6) supports the findings presented in Table 7.

## Discussion on results

In this study, we have predicted the renewable energy adoption intention of developing countries’ households, particularly wind energy. We have proposed eight hypotheses and analysed their influence on developing countries’ renewable energy adoption. For RQ1, we have presented H1, H1a, H2, H2a, and H8. For RQ2, we have proposed H3, H3a, H4, H4a, and H7. To answer RQ3, we have proposed H1b, H2b, H3b, and H4b, and in addition, we have analysed the direct relationship between cost value and social influence and REAI (H5 &H6). A summary of our proposed hypothesis results is presented in Table 9.

**Table 9:** Summary of results.

*Hypothesis*	*Path*	*Result*
H1	HC -> PBC	Supported
H1a	HC -> PBC -> REAI	Supported
H1b	SI x HC -> PBC -> REAI	Supported
H2	ARE -> PBC	Not supported
H2a	ARE -> PBC -> REAI	Not supported
H2b	SI x ARE -> PBC -> REAI	Supported
H3	EC -> ATE	Not supported
H3a	EC -> ATE -> REAI	Not supported
H3b	SI x EC -> ATE -> REAI	Not supported
H4	EK -> ATE	Supported
H4a	EK -> ATE -> REAI	Supported
H4b	SI x EK -> ATE -> REAI	Not supported
H5	CV -> REAI	Supported
H6	SI -> REAI	Supported
H7	ATE -> REAI	Supported
H8	PBC -> REAI	Supported

For RQ1, the results of this study revealed that HC influences the perceived behavioural control and perceived behavioural control also mediates the influence of HC on renewable energy adoption, but awareness of renewable energy (H2 & H2a) does not influence the PBC, neither the PBC mediates the relationship of ARE and REAI, but PBC has a significant direct effect on REAI. This means that the health-conscious attitude of the household shapes household perceived behaviour control that stimulates adoption intentions for renewable energy. This is consistent with earlier findings in the 5G adoption, where health consciousness proved to be a significant, influential factor [18] and to buy eco-friendly products [17]. Contrary, ARE is not a significant factor in our study. The possible reason can be that households are unaware of renewable energy advantages and their potential or are not open to experiencing the new technology at the domestic level and want to rely on national grids.

For RQ2, the study results revealed that households are not concerned about environmental changes and are not very eager to help in environmental change, sustainability, and improvement. Still, their knowledge about the environment significantly influences their attitude toward the environment and their renewable energy adoption intention. Attitude towards the environment supported by environmental knowledge also shapes the relationship dimension between EK and REAI. Hence if the households are well educated about the environment and related issues, they are likely to act responsibly and participate as a serious member of the community in collaborative actions against environmental sustainability and green energy sources. It is consistent with earlier researchers’ findings in a different context [17, 18, 32].

In response to RQ3, we have revealed that as much as households have social influence, their relationship of health consciousness PBC and REAI decreases, but the increase in social influence increases the awareness of renewable energy products and their influence on renewable energy adoption. It is in line with the findings of a recent study [7]. But on the other hand, social influence as a moderator does not have any influential effect on the relationship of EK and EC on ATE towards REAI. It contradicts the findings of the study that claim social networks significantly play their role as predictors and shape consumers’ behaviour towards biogas [7].

Lastly, we have observed that social influence and cost value directly influence the adoption intentions of renewable energy. It means that individuals are influenced by society and peers in their decision to adopt renewable energy. Although cost is a significant factor and has positive influence means, individuals are willing to pay for renewable energy installation units when they are aware of and compare the potential cost and advantages of renewable energy. Hence if consumers are educated carefully about the benefits of single-time installation cost and the environmental benefits of renewable energy, they seem serious about investing in installing renewable energy units.

### Theoretical contribution

The TPB has been the subject of many studies over the last several decades, with researchers elucidating its practical uses and explaining its theoretical foundations [65]. In the TPB, consumers are assumed to be rational agents whose decisions are guided by a set of predetermined criteria. These considerations serve as a foundation for selecting choices. Since it incorporates intangibles, the theory is seen as an improvement over prior models of customer behaviour. The TPB’s capacity to help customers in many different areas has earned it widespread acclaim worldwide. This research contributes to the TPB by expanding its theoretical framework with the introduction of three new dimensions environmental concern, attitude towards the environment, and the moderated mediation influence of social influence and proposed mediated relationships that may affect consumers’ propensity to embrace certain goods and technologies. These important theoretical contributions to the literature have never been addressed in any previously published work.

More than that, the current study contributes to previous research. For instance, it has been revealed that socially influenced households’ health consciousness towards adopting renewable energy decreases and awareness increases. Attitude toward the environment established by environmental knowledge and concern positively influences renewable energy adoption. The value of rigorous approaches and ideas is growing. Changes made with the help of the TPB will improve our understanding of these evolving methods based on well-established techniques [66].

Our study provides valuable information that legislators, academics, and practitioners may use. The TPB used in this investigation has certain innovative aspects that make it a useful tool for decision-making. This study uses unique dimensions to capture strategic goals that may be used for renewable energy selection and stakeholder/consumer education. Practitioners, legislators, and academics may use these criteria to allocate resources, rate projects, and choose which projects to pursue.

### Managerial implications

Our analysis provides the basis for the management policy recommendations, all of which aim to expand the use of renewable energy in developing countries in general and specifically in Pakistan. There is an essential need for time to prioritize the strategy of developing countries in overcoming the energy shortfall [8].

According to the research results, renewable energy may be rather pricey in developing countries. Governments and other authorities need to invest in technological advancements that reduce the cost of installation so that low-income households also can afford it and the burden on the national grid is reduced. Enhancing R&D efforts toward creating reasonably priced renewable energy that locals may adopt is a priority. The rate of renewable energy adoption may be accelerated by policies put in place by the government that are positive toward the technology.

In addition, provincial and federal governments need to introduce interest-free investment plans or encourage public-private partnerships to start small units to utilise the wind potential in the country and overcome the energy shortfall as public-private investment policy provided significant in Europe [6]. In addition, a decrease in the tax burden imposed on international renewable energy plant providers has the potential to entice global businesses to establish operations in Pakistan. The push of competition in the market helps to enhance product quality and ensure that customers have access to renewable energy that is beneficial to their wallets. When customers consider the benefits and drawbacks offered by various energy technology forms, they can choose the most suitable alternative from among the numerous possibilities. This will result in more healthy competition among the many renewable energy plant suppliers.

Consumers should be informed about the technology and its benefits via awareness efforts launched by the Ministry of Climate Change and other national and international stakeholders such as the UN (SDG7), such as seminars and discussion programs. Consumers will have a better positive impression of renewable energy and be more likely to utilise them as a consequence.

People in Pakistan do not have adequate information and comprehension of renewable energy. As suggested by earlier work, a lack of information regarding the advantages of new technology may lead to broad resistance to its usage [5]. As a result, all the marketing and promotion work that is done needs to be geared toward effectively disseminating renewable energy. This will have a positive impact on both the public perception of renewable energy and its adoption rates. There must be no room for manipulation inside the system in order to facilitate the further expansion of renewable energy. It is essential that regulations be drafted so that each renewable energy programme includes an adequate number of checks and balances. It is recommended that the powers of bureaucracy be reduced and that a proper tax structure be developed, with the stipulation that entrepreneurs and prosumers be provided with a tax break for the establishment of off-grid renewable energy projects.

Social campaigns and healthy debates on the use of renewable energy and related benefits to the national economy and ecological system need to encourage at a local level so that people join hands with the government and sustainable energy and development of developing countries can be achieved as in the case of biogas it has revealed in china [7].

## Conclusion

Based on the TPB, we have proposed a model that helps understand renewable energy adoption in developing countries at the domestic level. We have concluded that the health consciousness of domestic users in developing countries shapes their perceived behavioural control and environmental knowledge shapes their attitude towards the environment. In addition, PBC and ATE act as mediators to enhance the role of Health consciousness and environmental knowledge in adopting renewable energy. We have also observed that Higher social impact reduces PBC between health awareness and renewable energy uptake in socially affected families. But socially affected families are aware of renewable energy, and PBC between ARE and Renewable energy adoption rises with social influence.

We also conclude that social influence, cost value, PBC, and ATE positively influence domestic users to adopt renewable energy. Understanding the domestic users’ adoption intentions toward renewable energy can enhance the economic dependency of developing countries on renewable energy resources. The installation of renewable energy plants can help developing countries utilise their renewable energy resources, eventually cutting off the import of crude oil and the emission of greenhouse gases. Consequently, the dwindling economies can be stabilised, and the world can take steps to cope with global warming.

### Limitations

The study offers helpful insights for stakeholders and industry professionals; however, it does have a few limitations that have to be taken into account in any future research that is conducted. Firstly we have collected our sample from Sukkur, which is rich in wind energy. These sample selections or perceptions and awareness of coastal areas or other wind energy-rich areas can vary. Secondly, although we have collected respondents’ income, age, and education level, we did not study these demographics’ extensive role as barriers or facilitators in renewable energy adoption. Another limitation of the research is that we did not conduct an analysis of variance [67] test to determine whether or not there are significant variations between the means of independent groups. Future researchers may incorporate this analysis to provide more relevant findings. Thirdly we have not studied the potential barrier that can restrict households from shifting to renewable energy instead of buying from the national grid; future studies can combine different barriers and provide a meaningful solution to the issue. Lastly, constructing a theoretical framework may use various conceptual frameworks, such as theories and models. The fact that TPB served as the basis for the formulation of the research framework is one of the limitations of the present investigation. Future research may overcome this constraint by drawing inspiration from several other ideas.

## Supporting information

S1 Dataset (XLSX)

S1 File Supporting information- contains all the supporting tables. (DOCX)
