# Supplementary material for: A moderated mediation model to predict the adoption intention of renewable wind energy in developing countries
Source: PLoS One. 2023 Mar 2;18(3):e0281963. doi: 10.1371/journal.pone.0281963 (PMC9980790; doi:10.1371/journal.pone.0281963)
Supplement: S1 File — (DOCX) [file pone.0281963.s002.docx]

**Table 1. Cross loadings**

|  | ***ARE*** | ***ATE*** | ***CV*** | ***EC*** | ***EK*** | ***HC*** | ***PBC*** | ***REAI*** | ***SI*** |
| --- | --- | --- | --- | --- | --- | --- | --- | --- | --- |
| ARE1 | 0.883 | -0.07 | 0.71 | -0.05 | -0.044 | 0.413 | 0.414 | 0.537 | 0.668 |
| ARE2 | 0.894 | -0.049 | 0.656 | -0.028 | -0.031 | 0.352 | 0.422 | 0.532 | 0.624 |
| ARE3 | 0.846 | 0.028 | 0.539 | 0.01 | 0.026 | 0.369 | 0.359 | 0.441 | 0.575 |
| ARE4 | 0.914 | -0.053 | 0.607 | -0.068 | -0.067 | 0.4 | 0.405 | 0.517 | 0.65 |
| ATE1 | -0.057 | 0.898 | -0.036 | 0.372 | 0.601 | 0.002 | -0.052 | 0.003 | -0.053 |
| ATE2 | -0.05 | 0.918 | -0.025 | 0.376 | 0.621 | 0.011 | -0.014 | 0.024 | -0.042 |
| ATE3 | -0.031 | 0.932 | -0.009 | 0.393 | 0.604 | 0.024 | -0.023 | 0.027 | -0.032 |
| ATE4 | -0.046 | 0.934 | -0.025 | 0.401 | 0.609 | 0.038 | -0.044 | 0.013 | -0.035 |
| ATE5 | -0.014 | 0.912 | -0.007 | 0.397 | 0.604 | 0.039 | -0.054 | 0.017 | -0.004 |
| CV1 | 0.508 | -0.004 | 0.777 | -0.027 | 0.017 | 0.381 | 0.357 | 0.403 | 0.487 |
| CV2 | 0.633 | -0.042 | 0.879 | -0.055 | -0.024 | 0.362 | 0.387 | 0.491 | 0.561 |
| CV3 | 0.668 | -0.008 | 0.894 | -0.004 | 0.062 | 0.449 | 0.399 | 0.522 | 0.583 |
| EC1 | -0.047 | 0.353 | -0.029 | 0.813 | 0.376 | -0.054 | 0.023 | 0.002 | 0.007 |
| EC2 | -0.068 | 0.396 | -0.071 | 0.846 | 0.409 | -0.107 | -0.016 | -0.04 | -0.035 |
| EC3 | -0.014 | 0.364 | -0.008 | 0.845 | 0.404 | 0.02 | 0.036 | 0.068 | 0.027 |
| EC4 | -0.025 | 0.294 | -0.011 | 0.824 | 0.347 | -0.008 | 0.009 | 0.006 | 0.026 |
| EC5 | -0.006 | 0.329 | -0.009 | 0.822 | 0.352 | 0.057 | 0.082 | 0.062 | 0.019 |
| EK1 | -0.079 | 0.609 | -0.021 | 0.397 | 0.851 | -0.023 | -0.067 | 0.001 | -0.057 |
| EK2 | -0.095 | 0.611 | -0.05 | 0.394 | 0.861 | -0.015 | -0.058 | -0.013 | -0.073 |
| EK3 | 0.015 | 0.513 | 0.074 | 0.379 | 0.862 | 0.033 | -0.005 | 0.061 | 0.034 |
| EK4 | 0.03 | 0.511 | 0.069 | 0.386 | 0.846 | 0.062 | -0.011 | 0.078 | 0.042 |
| EK5 | 0.001 | 0.544 | 0.044 | 0.382 | 0.823 | 0.023 | -0.035 | 0.049 | 0.004 |
| HC1 | 0.239 | 0.033 | 0.295 | -0.029 | 0.006 | 0.762 | 0.401 | 0.473 | 0.29 |
| HC2 | 0.312 | -0.04 | 0.324 | -0.051 | 0.009 | 0.754 | 0.405 | 0.459 | 0.343 |
| HC3 | 0.338 | 0.092 | 0.33 | -0.025 | 0.031 | 0.785 | 0.475 | 0.628 | 0.343 |
| HC4 | 0.436 | -0.012 | 0.483 | 0.016 | 0.004 | 0.808 | 0.503 | 0.588 | 0.452 |
| PBC1 | 0.322 | -0.052 | 0.309 | -0.026 | -0.018 | 0.411 | 0.711 | 0.434 | 0.295 |
| PBC2 | 0.341 | -0.035 | 0.321 | -0.006 | -0.05 | 0.46 | 0.767 | 0.524 | 0.261 |
| PBC3 | 0.378 | -0.043 | 0.37 | 0.068 | -0.062 | 0.412 | 0.809 | 0.642 | 0.342 |
| PBC4 | 0.354 | 0 | 0.373 | 0.046 | -0.005 | 0.495 | 0.792 | 0.629 | 0.32 |
| REAI1 | 0.549 | 0.024 | 0.535 | 0.02 | 0.025 | 0.646 | 0.697 | 0.899 | 0.488 |
| REAI2 | 0.481 | 0.016 | 0.45 | 0.008 | 0.037 | 0.607 | 0.653 | 0.889 | 0.421 |
| REAI3 | 0.511 | 0.008 | 0.51 | 0.03 | 0.041 | 0.62 | 0.614 | 0.901 | 0.501 |
| SI1 | 0.673 | -0.064 | 0.591 | -0.011 | -0.039 | 0.41 | 0.358 | 0.473 | 0.87 |
| SI2 | 0.643 | -0.027 | 0.574 | 0.051 | -0.025 | 0.419 | 0.35 | 0.463 | 0.887 |
| SI3 | 0.637 | -0.033 | 0.58 | -0.013 | 0.003 | 0.452 | 0.366 | 0.489 | 0.895 |
| SI4 | 0.554 | 0.001 | 0.509 | 0.004 | 0.006 | 0.35 | 0.318 | 0.423 | 0.876 |

**Table 2. Total Variance Explained**

| Component | Initial Eigenvalues | | | Extraction Sums of Squared Loadings | | |
| --- | --- | --- | --- | --- | --- | --- |
|  | Total | % of Variance | Cumulative % | Total | % of Variance | Cumulative % |
| 1 | 3.800 | 42.227 | 42.227 | 3.800 | 42.227 | 42.227 |
| 2 | 2.016 | 22.401 | 64.629 | 2.016 | 22.401 | 64.629 |
| 3 | .919 | 10.207 | 74.836 |  |  |  |
| 4 | .664 | 7.380 | 82.216 |  |  |  |
| 5 | .449 | 4.993 | 87.209 |  |  |  |
| 6 | .361 | 4.009 | 91.218 |  |  |  |
| 7 | .346 | 3.842 | 95.060 |  |  |  |
| 8 | .249 | 2.767 | 97.826 |  |  |  |
| 9 | .196 | 2.174 | 100.000 |  |  |  |
| Extraction Method: Principal Component Analysis. | | | | | | |
